# Therapeutic effects of polydeoxyribonucleotide in an in vitro neuronal model of ischemia/reperfusion injury

**DOI:** 10.1038/s41598-023-32744-9

**Published:** 2023-04-12

**Authors:** Seongmoon Jo, Ahreum Baek, Yoonhee Cho, Sung Hoon Kim, Dawoon Baek, Jihye Hwang, Sung-Rae Cho, Hyun Jung Kim

**Affiliations:** 1 Department and Research Institute of Rehabilitation Medicine, Yonsei University College of Medicine, Seoul, South Korea; 2 Brain Korea 21 PLUS Project for Medical Science, Yonsei University College of Medicine, Seoul, South Korea; 3 Department of Rehabilitation Medicine, Yonsei University Wonju College of Medicine, Wonju, South Korea; 4 Department of Medicine, Yonsei University College of Medicine, Seoul, South Korea; 5 Graduate Program of Biomedical Engineering, Yonsei University College of Medicine, Seoul, South Korea; 6 Rehabilitation Institute of Neuromuscular Disease, Yonsei University College of Medicine, Seoul, South Korea; 7 Department of Rehabilitation Medicine, Nowon Eulji Medical Center, Eulji University School of Medicine, Seoul, South Korea

**Keywords:** Cell biology, Neuroscience

## Abstract

Polydeoxyribonucleotide (PDRN) is an agonist that selectively stimulates adenosine A_2A_ receptor (ADORA2A), which suppresses inflammatory responses. Ischemia/reperfusion (I/R) injury plays a major role in the pathogenesis of ischemic stroke by inducing neuroinflammation. Therefore, this study aimed to investigate the therapeutic effects of PDRN in an in vitro I/R injury model. The in vitro model was established with differentiated Neuro-2a cells under oxygen and glucose deprivation condition. The cells were treated with PDRN for 24 h under reoxygenation condition. As the results of RNA-seq transcriptome analysis, CSF1, IL-6, PTPN6, RAC2, and STAT1 were identified of its relation to the effect of PDRN on inflammatory responses in the model. To further investigate therapeutic effects of PDRN, RT-qPCR, western blotting, LDH assay, and TUNEL assay were performed. PDRN significantly reversed the expression of genes and proteins related to inflammatory responses. The elevated ADORA2A expression by PDRN treatment downregulated JAK/STAT pathway in the model. Furthermore, PDRN inhibited neuronal cell death in the model. Consequently, our results suggested that PDRN alleviated inflammatory responses through inhibition of JAK/STAT pathway by mediating ADORA2A expression and inhibited neuronal cell death in the model. These results provide significant insights into potential therapeutic approaches involving PDRN treatment for I/R injury.

## Introduction

One of the common features of ischemic stroke is ischemia–reperfusion injury (I/R injury), and I/R injury in the brain causes serious irreversible damage to neurons^1,2^. I/R injury occurs when the blood flow through cerebral blood vessels is restored after a period of ischemia with the reintroduction of oxygen and glucose upon reperfusion of ischemic tissue that triggers the oxidative stress, initiating a series of pathological I/R injuries^3,4^. A cascade of I/R injuries leads to the death of cells and tissues and develops lesions with upregulated immune responses^3,5^. Therefore, it is necessary to protect neuronal cells from irreversible damage and alleviate the inflammatory responses by administering appropriate treatments for I/R injury; however, there is a lack of ideal therapeutics against I/R injury^2,3^.

Polydeoxyribonucleotide (PDRN), a DNA-derived drug, is a mixture of 50–1500 kDa deoxyribonucleotides^6^. These DNA fragments are obtained by extracting sperm from two types of salmon, *Oncorhynchus mykiss* and *Oncorhynchus keta*, and undergo specific purification and sterilization processes^7,8^. PDRN is known for its ability to selectively stimulate adenosine A_2A_ receptor (ADORA2A), leading to the regulation of inflammation, ischemia, angiogenesis, growth factor stimulus, and apoptosis in various diseases^6,9–13^. Regardless of these pharmacological effects of PDRN, there were few studies regarding the therapeutic effects on I/R injury.

Therefore, this study aimed to elucidate the therapeutic effects and mechanisms of PDRN on an in vitro I/R injury model. In the current study, we used oxygen and glucose deprivation/reoxygenation (OGD/R) condition to establish mimic I/R injured neurons of ischemic stroke. OGD/R in vitro model is widely used to replicate the cellular pathophysiology of in vitro ischemic stroke system^14^. Thus, we conducted experimental studies on the in vitro I/R injury model to investigate the potential therapeutic effects of PDRN on I/R injury.

## Materials and methods

### Cell culture

The Neuro-2a (N2a) cells, derived from mouse neuroblastoma, were purchased from the American Type Culture Collection (Manassas, VA, USA). N2a cells exhibit properties of neuronal stem cells and can differentiate into neuronal cells when treated with 20 μM retinoic acid (RA)^1,15^. The cells were incubated in culture dishes into Dulbecco’s modified Eagle’s medium (DMEM; Hyclone, Logan, UT, USA) with 10% fetal bovine serum (FBS; Serum Source International, Charlotte, NC, USA) and 1% penicillin/streptomycin (Gibco, Rockville, MD, USA) in a humidified 5% CO_2_ atmosphere at 37°C. When the cells reached 70–80% of confluency, the medium was replaced as a differentiation medium, which contained 2% FBS and 20 μM RA in DMEM for four days. Differentiated N2a cells were maintained in a humidified atmosphere of 5% CO_2_ at 37°C, and the differentiation medium was changed every two days.

### Oxygen and glucose deprivation/reoxygenation (OGD/R) and drug treatment

**Figure 1:**
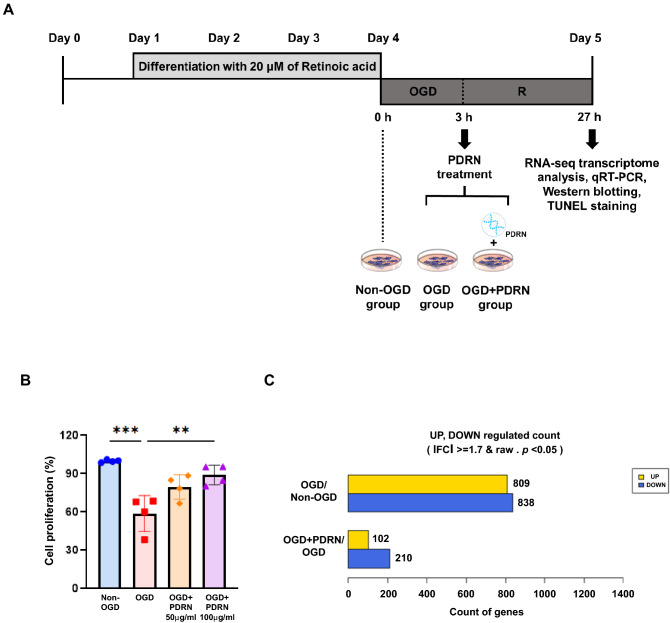
Effect of PDRN on an in vitro I/R injury model. (**A**) Experimental design of PDRN treatment on an in vitro I/R injury model. (**B**) Effect of PDRN on the cell viability of in vitro I/R injury model. (**C**) Bar graphs show the number of differentially expressed genes in OGD group compared with Non-OGD group and in OGD + PDRN group compared with OGD group. Values are presented as means ± standard error of the mean (SEM). Statistically significant differences are shown as **p < 0.01, ***p < 0.001.

The following procedures were adapted to establish an in vitro I/R injury model from previous studies^1,16–18^. Differentiated N2a cells were washed three times with phosphate-buffered saline (PBS), and the medium was replaced with deoxygenated, glucose-free balanced salt solution (Gibco) in hypoxic condition (O_2_ tension 1%) for 3 h. Following OGD condition, injured cells were replaced onto the growth medium. PBS and 50 or 100 μg/ml PDRN (Placentex Integro, Mastelli Srl, Italy) was added to the growth medium of injured cells for 24 h according to the following experimental groups. Cells treated with OGD and PBS were classified as the OGD group, and cells treated with OGD and PDRN were classified as the OGD + PDRN group. Differentiated N2a cells without OGD were classified as the non-OGD group (Fig. 1A).

### Cell proliferation

To analyze the proliferation of the in vitro I/R injury model, the number of cells in the non-OGD, OGD, and OGD + PDRN groups were calculated using an advanced detection and accurate measurement (ADAM) automatic cell counter (NanoEnTek Inc., Seoul, South Korea).

### RNA preparation

After the cellular experiments, total RNA was isolated from the cells of all the groups with TRIzol reagent (Thermo Fisher Scientific, Waltham, MA, USA) was used for RNA isolation^19^ according to the manufacturer’s instructions. A NanoDrop spectrophotometer (Thermo Fisher Scientific) was used to confirm RNA quantity and purity.

### RNA-sequencing (RNA-seq) transcriptome array

RNA-seq transcriptome array of the non-OGD, OGD, and OGD + PDRN groups was performed at Macrogen Inc. (Seoul, Korea) with the HiSequation 2000 platform (Illumina, San Diego, CA, USA) according to methods detailed in our previous study^20^.

### RNA-sequencing transcriptome analysis

In this study, fold change (FC) criteria (FC ≥|1.7|) were used to identify the differentially expressed genes (DEGs) from the results of RNA-seq transcriptome array. To identify their roles, three different pairs of DEGs were submitted to the Database for Annotation, Visualization and Integrated Discovery (DAVID) v.6.8 annotation tool^21^.

### Quantitative real-time reverse transcription-polymerase chain reaction

RT-qPCR was performed to validate the transcriptome analysis results. ReverTra Ace ® qPCR RT Master Mix with gDNA Remover (Toyobo, Osaka, Japan) was used to prepare the cDNA from total RNA, according to the manufacturer’s instructions. RT-qPCR was performed to measure the mRNA levels of the genes of interest using qPCRBIO SyGreen Mix Hi-ROX (PCR BIOSYSTEMS, London, UK) on a StepOnePlus Real-Time PCR System (Applied Biosystems, Foster City, CA, USA). The 2^−ΔΔCT^ method was used for data analysis^22^. Supplementary Table S1 lists the primers used for RT-qPCR.

### Western blotting

The proteins were extracted from the cell pellets. Proteins were boiled for 10 min and loaded onto 4–12% bis Tris gels (Invitrogen, Waltham, MA, USA) for separation. The separated proteins were then blotted onto polyvinylidene difluoride (PVDF) membranes (Invitrogen) with 20% (v/v) methanol in NuPage Transfer Buffer (Invitrogen) at 15 V for 4 h at 4°C. Tris-buffered saline containing 0.01% Tween 20 (TBST) in 5% skim milk (Difco, BD Biosciences, Oxford, UK) was used to block the membranes for 1 h. The blots were washed three times with TBST for 10 min and then incubated overnight at 4 °C with primary antibodies specific to the following target proteins: phosphorylated JAK1, phosphorylated JAK2, phosphorylated STAT1 (1:1000; Cell Signaling Technology, Cambridge, UK), CSF1, IL-6, PTPN6, RAC2, TNFα, IL-1α, IL-1β, phosphorylated STAT3, STAT3 ADORA2A, JAK1, JAK2, STAT1, SOCS3, Bax, Bcl-2, and β-actin (1:1000; Santa Cruz Biotechnology, Santa Cruz, CA, USA). After incubation, the blots were washed thrice with TBST and incubated for 1 h with a horse-radish peroxidase–conjugated secondary antibody (1:3000; Santa Cruz) at 25°C. Finally, the blots were visualized using an enhanced chemiluminescence detection system (Amersham Pharmacia Biotech, Little Chalfont, UK).

### Lactate dehydrogenase assay

To analyze neuronal cell death, N2a cells were seeded on a 96-well cell culture plate (SPL Life Sciences, Gyeonggi-do, Korea) and OGD and PDRN treatments were performed as previously described above. The death of the in vitro I/R injury model was evaluated using a cytotoxicity lactate dehydrogenase (LDH) assay kit (Dojindo, Kumamoto, Japan) according to the manufacturer's protocol. Briefly, 10 µl of lysis buffer was added to each well and the cells were cultured at 37°C in CO_2_ for 30 min. A total of 100 µL of the working solution was then added to each well, and the samples were cultured at room temperature in the dark. Stop solution (50 µl) was then added to each well, and LDH levels in the culture supernatant were analyzed immediately by measuring the absorbance at 490 nm using a microplate reader (VersaMax, Molecular Devices, San Jose, CA, USA).

### Terminal dUTP nick end-labeling assay

To analyze apoptosis, N2a cells were seeded on a cell culture slide (SPL Life Sciences, Gyeonggi-do, Korea) and OGD and PDRN treatments were performed as previously described above. The DeadEnd™ Fluorometric TUNEL System (Promega Madison WI USA) was used to assess apoptosis according to the manufacturer’s protocol. Briefly, the samples were mounted on glass slides with a fluorescent mounting medium with DAPI for imaging using an LSM 700 fluorescence microscope (Carl Zeiss, Gottingen, Germany). The number of positively stained cells over the total number of cells per specimen field was measured, and the percentage of positive cells was calculated. Four individual specimens were analyzed per group.

### Statistical analysis

All data are expressed as the mean ± standard error of the mean (SEM), and all experiments were repeated at least four times with four technical replicates in each group. The Statistical Package for Social Sciences v.25.0 (IBM Corp. Released 2015. IBM SPSS Statistics for Windows, v.25.0. Armonk, NY, USA) was used for the statistical analyses. The significance of intergroup differences was estimated using Student’s paired *t*-test or one-way analysis of variance (ANOVA). Statistical significance was set at *p* < 0.05.

## Results

### PDRN attenuated the suppression of cell proliferation in an in vitro I/R model

To investigate the effects of PDRN against ischemic stroke, an in vitro I/R injury model was established using N2a cells (Fig. 1A). The proliferation rate of cells treated with PDRN at different concentrations (50 and 100 μg/ml) was analyzed. The results showed that the cell proliferation rate of the OGD group was significantly reduced compared with that of the non-OGD group (Fig. 1B). After 24 h of treatment with different concentrations of PDRN, the cell proliferation rate of the 50 μg/ml OGD + PDRN group was not significantly different from that of the OGD group (Fig. 1B). However, in the 100 μg/ml OGD + PDRN group, the cell proliferation rate was significantly increased compared to that in the OGD group (Fig. 1B). These results indicated that PDRN at 100 μg/ml concentration protected against OGD-induced cell death in the in vitro I/R injury model. The 100 μg/ml concentration of PDRN was selected for the subsequent experiment.

### Inflammation-related genes were differentially expressed in an in vitro I/R model after PDRN treatment

**Figure 2:**
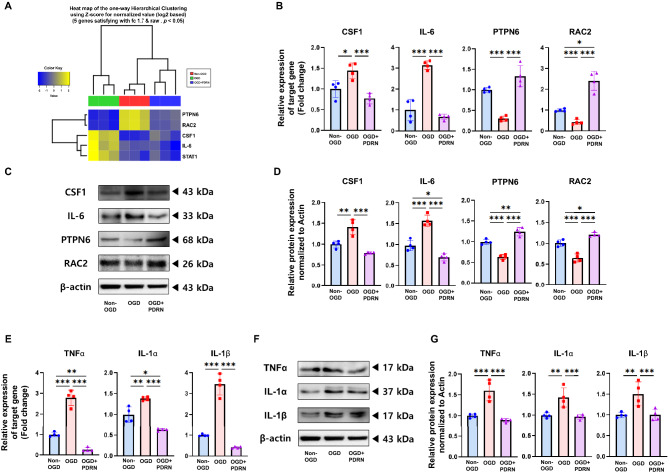
Effect of PDRN on genes related to the inflammation of in vitro I/R injury model. (**A**) Heat map representing effect of PDRN treatment on 5 genes related to the inflammatory response of in vitro I/R model. (**B**) The relative gene expression of CSF1, IL-6, PTPN6, and RAC2 in Non-OGD, OGD, and OGD + PDRN groups detected by RT-qPCR. (**C**) Western blotting analysis of CSF1, IL-6, PTPN6, and RAC2 in Non-OGD, OGD, and OGD + PDRN groups (**D**) Quantification of western blotting signals for CSF1, IL-6, PTPN6, and RAC2. (**E**) The relative gene expression of TNFα, IL-1α, and IL-1β in Non-OGD, OGD, and OGD + PDRN groups detected by RT-qPCR. (**F**) Western blotting analysis of TNFα, IL-1α, and IL-1β in Non-OGD, OGD, and OGD + PDRN groups (**G**) Quantification of western blotting signals for TNFα, IL-1α, and IL-1β. Values are presented as means ± SEM. Statistically significant differences are shown as *p < 0.05, **p < 0.01, ***p < 0.001.

To identify the DEGs following treatment with PDRN in an in vitro I/R injury model, we performed transcriptome analysis using RNA-seq transcriptome array. A total of 1,959 DEGs were identified using two pairwise comparisons. Comparing the OGD (OGD, 3 h) and non-OGD groups, 809 transcripts exhibited expression higher than 1.7-fold, and 838 transcripts exhibited expression lower than −1.7-fold. In the comparison between the OGD + PDRN (OGD, 3 h and PDRN, 24 h) and OGD groups, 102 transcripts exhibited an expression higher than 1.7-fold, and 210 transcripts exhibited an expression lower than −1.7-fold (Fig. 1C, Supplementary Fig. 1). We analyzed these individual DEGs and identified genes related to inflammation since PDRN is well-known as an anti-inflammatory drug used in the treatment of several diseases, such as osteoarthritis, fasciitis, mucositis, and tendinopathy^20,23–25^. The identified five genes have been listed with the respective fold changes (Table 1). CSF1, IL-6, and STAT1, which are related to the pro-inflammatory mechanism, were significantly upregulated in the OGD group compared to the non-OGD group. However, PTPN6 and RAC2, related to the anti-inflammatory mechanism, were significantly downregulated in the OGD group compared to the non-OGD group. In contrast, CSF1, IL-6, and STAT1 were significantly downregulated, and PTPN6 and RAC2 were significantly upregulated in the OGD + PDRN group compared to the OGD group (Table 1, Fig. 2A).

**Table 1:** Expression levels of inflammation-related differentially expressed genes between the OGD and non-OGD groups and the OGD and OGD + PDRN groups.

Gene Symbol	OGD/non-OGD (Fold change)	OGD + PDRN/OGD (Fold change)
CSF1	2.488905	–1.882492
IL-6	2.425789	–3.318641
STAT1	1.742832	–1.765480
PTPN6	–4.701431	1.802272
RAC2	–7.991739	1.877436

CSF1, colony-stimulating factor 1; IL-6, interleukin 6; STAT1, signal transducer and activator of transcription 1; PTPN6, protein tyrosine phosphatase non-receptor type 6; RAC2, Rac family small GTPase 2.

### PDRN regulated inflammation-related factors in an in vitro I/R injury model

RT-qPCR and western blotting were performed to validate the effect of PDRN on inflammation in the in vitro I/R injury model. The RT-qPCR results showed that the expression of CSF1 and IL-6 was significantly elevated in the OGD group compared to that in the non-OGD group. However, the expression of the genes in the OGD + PDRN group was significantly downregulated than that in the OGD group. The expression of the genes related to anti-inflammatory mechanisms, such as PTPN6 and RAC2, was significantly downregulated in the OGD group than that in the non-OGD group. In contrast, the expression of these genes in the OGD + PDRN group was significantly elevated compared to that in the OGD group (Fig. 2A, B). Western blotting analysis showed that the protein expression levels of CSF1 and IL-6 in the OGD group were significantly upregulated than that in the non-OGD group. Meanwhile, the protein expression levels of CSF1 and IL-6 were significantly decreased in the OGD + PDRN group than that in the OGD group. The protein expression level of PTPN6 and RAC2 in the OGD group was significantly decreased than that in the non-OGD group. In the OGD + PDRN group, the protein expression levels of PTPN6 and RAC2 were significantly upregulated than that in the OGD group (Fig. 2C, D). Moreover, we investigated the other inflammatory mediators, namely, TNFα, IL-1α, and IL-1β, which are known to be related to I/R injury^26–28^. In the results, the gene and protein expression levels of TNFα, IL-1α, and IL-1β in the OGD group were significantly elevated than that in the non-OGD group. However, the gene and protein expression levels of TNFα, IL-1α, and IL-1β in the OGD + PDRN group were significantly downregulated compared to that in the OGD group (Fig. 2E–G). These results indicated that PDRN may regulate inflammation by elevating the expression of anti-inflammatory genes and decreasing the expression of pro-inflammatory genes in the *in-vitro* I/R injury model.

### PDRN suppressed inflammation in an in vitro I/R injury model via downregulation of the JAK/STAT pathway

**Figure 3:**
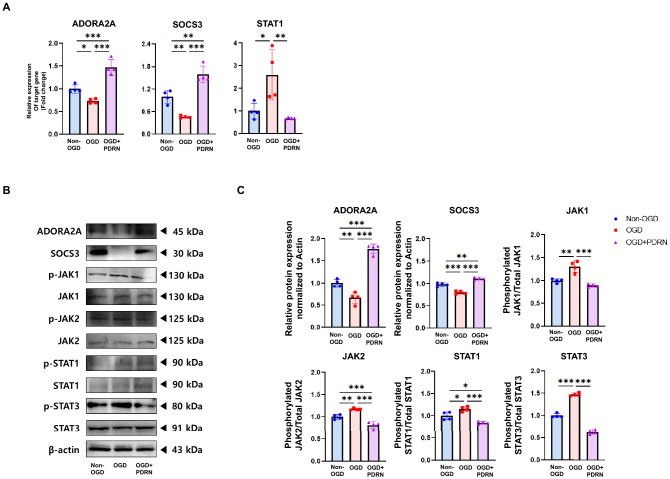
Effect of PDRN on JAK/STAT pathway of in vitro I/R Injury model. (**A**) The relative gene expression of ADORA2A, SOCS3, and STAT1 in Non-OGD, OGD, and OGD + PDRN groups detected by RT-qPCR. (**B**) Western blot analysis of the JAK/STAT pathway in Non-OGD, OGD, and OGD + PDRN groups (**C**) Quantification of western blotting signals for the JAK/STAT pathway. Values are presented as means ± SEM. Statistically significant differences are shown as *p < 0.05, **p < 0.01, ***p < 0.001.

PDRN is a well-known ADORA2A agonist, and activation of the A_2A_ receptor is related to the JAK/STAT pathway via induction of the inhibitory factor SOCS3^26,27^. RT-qPCR and western blotting were performed to investigate the effect of PDRN treatment on the JAK/STAT pathway in the in vitro I/R injury model. The results showed that the relative mRNA expression of the ADORA2A and SOCS3 was upregulated after treatment with PDRN (Fig. 3A). Moreover, the protein expression of the ADORA2A and SOCS3 was upregulated after treatment with PDRN (Fig. 3B, C). Furthermore, the levels of phosphorylated JAK1, JAK2, STAT1, and STAT3 in the OGD group were significantly increased compared to that in the non-OGD group, whereas the levels of phosphorylated JAK1, JAK2, STAT1, and STAT3 in the OGD + PDRN group decreased significantly as compared to that in the OGD group (Fig. 3B, C). The results indicated that PDRN treatment acted as ADORA2A agonist and downregulated JAK/STAT pathway by inducing the inhibitory protein SOCS3 in the in vitro I/R injury model.

### PDRN inhibited apoptosis in an in vitro I/R injury model

**Figure 4:**
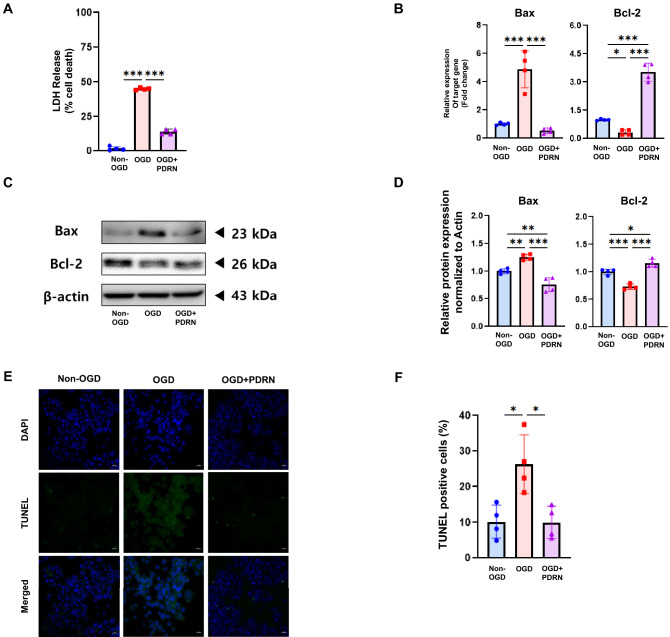
Effect of PDRN on apoptosis of in vitro I/R injury model. (**A**) The relative amount of released LDH detected by LDH assay (**B**) The relative gene expression of Bax and Bcl-2 detected by RT-qPCR (**C**) Western blotting analysis of Bax and Bcl-2 in Non-OGD, OGD, and OGD + PDRN groups (**D**) Quantification of western blotting signals of Bax and Bcl-2 (**E**) TUNEL assay in Non-OGD, OGD, and OGD + PDRN groups (**F**) Quantification of TUNEL assay. Values are presented as means ± SEM. Scale bars = 100 µm. Statistically significant differences are shown as *p < 0.05, **p < 0.01, ***p < 0.001.

To investigate whether PDRN inhibits neuronal death, the amount of released LDH, the expression of apoptosis-related proteins and genes, and the number of TUNEL-positive cells were examined. The amount of released LDH showed elevation in the OGD group compared to that in the non-OGD group. In the OGD + PDRN group, the relative level of released LDH was significantly downregulated compared to that in the OGD group (Fig. 4A). The expression of the pro-apoptotic gene Bax was significantly elevated, while the expression of anti-apoptotic gene Bcl-2 wassignificantly decreased in the OGD group compared to that in the non-OGD group. However, PDRN treatment significantly reversed these changes (Fig. 4B). The protein expression of Bax was significantly elevated, while the protein expression of Bcl-2 was significantly decreased in the OGD group compared to that in the non-OGD group. PDRN treatment significantly reversed these changes (Fig. 4C, D). Furthermore, the percentage of TUNEL-positive cells was significantly increased in the OGD group (Fig. 4E, F). However, the percentage of TUNEL-positive cells was significantly decreased after treatment with PDRN (Fig. 4E, F). Taken together, these results showed that PDRN treatment could protect neurons from neuronal cell death in an in vitro I/R injury model.

## Discussion

**Figure 5:**
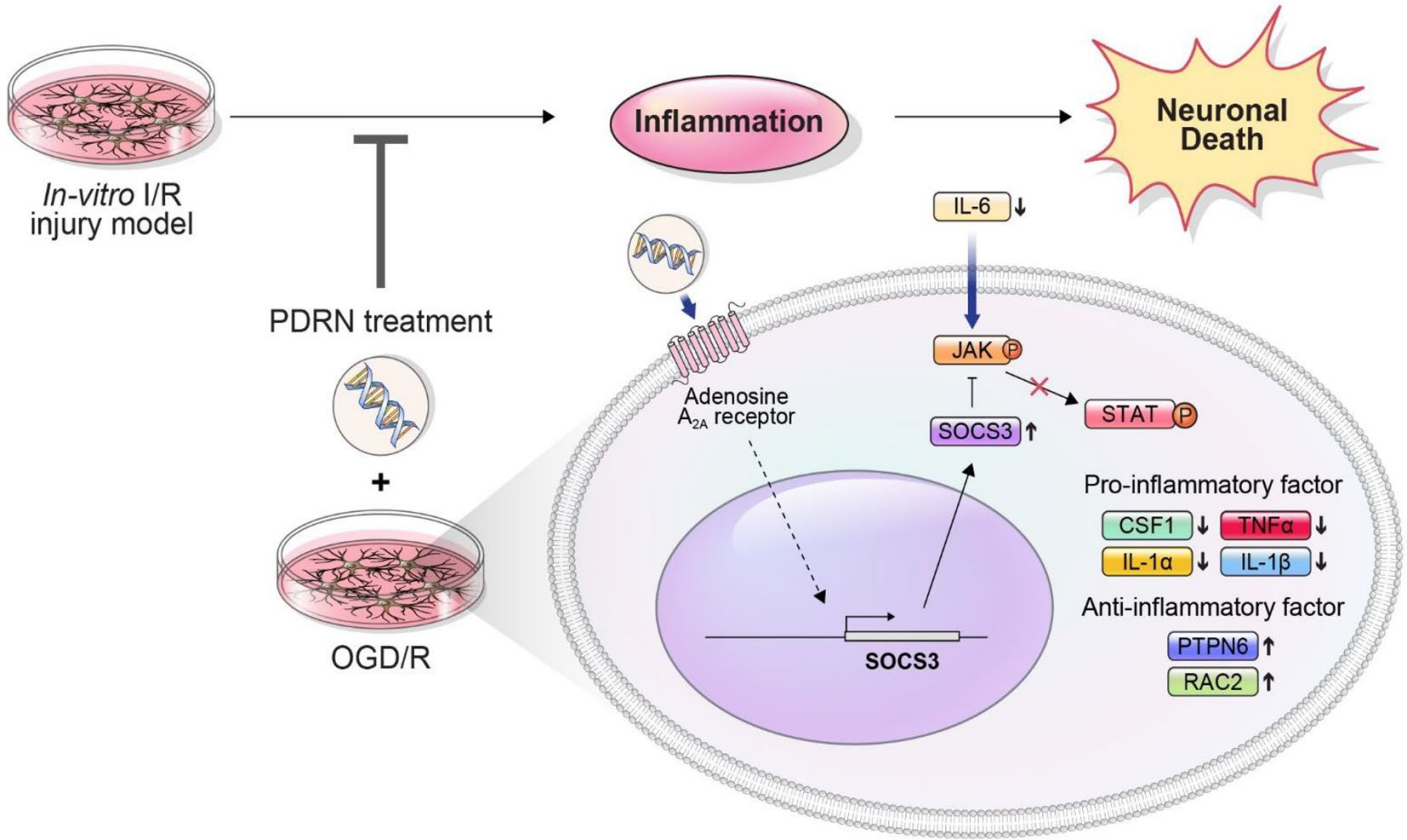
Schematic diagram of PDRN treatment on an in vitro I/R injury model for attenuating an inflammation via downregulation of the JAK/STAT pathway. Potential therapeutic mechanisms PDRN on the in vitro I/R injury model. PDRN could act as an agonist of ADORA2A in the in vitro I/R injury model. The activated ADORA2A upregulated the expression of SOCS3 and it suppressed JAK/STAT pathway. Moreover, PTPN6 and RAC2, which are anti-inflammatory factors, were increased, whereas CSF1, IL-6, TNFα, IL-1α, and IL-1β, which are pro-inflammatory factors were decreased after PDRN treatment.

The current study indicated that PDRN could decrease inflammatory responses via downregulation of JAK/STAT pathway by regulating ADORA2A expression and inhibit neuronal cell death in the in vitro I/R injury model (Fig. 5).

Ischemic brain injury triggers an increase in inflammatory responses by the accumulation of inflammatory cells and mediators and it could exacerbate the brain damage; thus, it is important to modulate the inflammatory responses to indirectly protect neurons and axons^28^. It is widely accepted that PDRN activates as an ADORA2A agonist and exhibits an effect of anti-inflammation^29^. In previous studies, ADORA2A agonists were administrated to reduce neuroinflammation by inhibiting pro-inflammatory mediators and protect brain tissue from cell death^30,31^. Moreover, ADORA2A activation is well known to elicit beneficial anti-inflammatory effects on ischemic models^32^. In our present study, we administrated PDRN as a treatment to increase the expression of ADORA2A on the in vitro I/R injury model. The results showed that PDRN suppressed the production of several pro-inflammatory cytokines such as CSF1, IL-6, TNFα, IL-1α, IL-1β and upregulated ani-inflammatory cytokines such as PTPN6, and RAC2 on the in vitro I/R injury model. These results indicated that PDRN treatment might have an effect on inflammatory responses in the in vitro I/R injury model.

Moreover, we investigated the effect of PDRN on the modulation of JAK/STAT pathway since there were some reports that stated that ADORA2A activation is related to the activation of JAK/STAT pathway^3,29^. JAK/STAT pathway contributes to the control of various physiological processes, which were involved in cell proliferation, differentiation, inflammation, and apoptosis^33^. As shown in the results, the expression of SOCS3, which is the main mediator between ADORA2A and JAK/STAT pathway, was upregulated after PDRN treatment on the in vitro I/R injury model. The upregulated SOCS3 induced to downregulate phosphorylation levels of JAK1, JAK2, STAT1, and STAT3 in the in vitro I/R injury model. These results demonstrated that PDRN could suppress inflammatory cascades by downregulating JAK/STAT pathway in the in vitro I/R injury model.

In addition, the activation of JAK/STAT pathway could trigger neuronal cell death in ischemic stroke injury^34^. In light of our results, we further investigated the effect of PDRN treatment on neuronal cell death by downregulating JAK/STAT pathway and inflammatory responses in the in vitro I/R injury model. The results showed that the amount of released LDH was downregulated after PDRN treatment. Moreover, the percentage of TUNEL-positive cells and Bax expression were suppressed and Bcl-2 expression was enhanced by PDRN treatment on the in vitro I/R injury model. These results indicated that PDRN could inhibit neuronal cell death in the in vitro I/R injury model.

This experimental study provides new insights into the therapeutic effects of PDRN on I/R injury. However, there are limitations to these findings. The limitations of this study are that mechanistic insight is only associative and lacks of functional and/or translational relevance. To reinforce the suggested interpretation of this study, it is imperative to clarify the detailed mechanisms underlying the protective actions of PDRN treatment with rescue experiments using an activator or inhibitor of JAK or STAT. Moreover, this study exclusively relied on the in vitro I/R injury model to examine the effects of PDRN treatment and lacked experimental data from animals or humans. Therefore, further basic and clinical research are essential to validate the anti-inflammatory responses of PDRN in in vivo I/R injury models and humans.

In conclusion, PDRN treatment suppressed inflammatory responses through ADORA2A-mediated downregulation of JAK/STAT pathway and inhibited neuronal death in the in vitro I/R injury model. Based on these findings, PDRN treatment may be useful in developing more reliable therapeutic strategies for patients with I/R injuries.

## Supplementary Information

Supplementary Information 1.

Supplementary Information 2.

Supplementary Information 3.

## Data Availability

RNA-Seq data used in this study are deposited in the NCBI database as the sequence read archive (SRA) format under the accession number PRJNA901365.
