# Supplementary material for: Therapeutic effects of polydeoxyribonucleotide in an in vitro neuronal model of ischemia/reperfusion injury
Source: Sci Rep. 2023 Apr 12;13:6004. doi: 10.1038/s41598-023-32744-9 (PMC10097812; doi:10.1038/s41598-023-32744-9)
Supplement: Supplementary file 2 — Supplementary Information 2. [file 41598_2023_32744_MOESM2_ESM.docx]

**Supplementary Table 1.** Primers used for quantitative real-time reverse transcription-polymerase chain reaction.

| **Gene symbol** | **Forward** primer (5′ → 3′) | **Reverse** primer (5′ → 3′) |
| --- | --- | --- |
| CSF1 | GCC ATC GGT GAC TTC CTC AA | TTG AAG TAG GTG TAG CGC GG |
| IL-6 | ACT ATG GCT ACC GCT TTG CC | AGG TTC ATG AGG ATG CGA GC |
| STAT1 | CAT CAG TAC CCT GGC CCT TG | ACT GCT CTT GCT CCC TTC AC |
| PTPN6 | CTG GGA CTC TGC ACA ACC TT | CAG TGT CGT GAT GGC GTA GA |
| RAC2 | AGC ACG GAA AGA GAG ACA GC | GCT GTC ATT CTG GGA CCT GT |
| TNFα | CCC TCA CAC TCA CAA ACC AC | ACA AGG TAC AAC CCA TCG GC |
| IL-1α | CAC AGG TAG TGA GAC CGA CC | CGA CTT TGT TCT TTG GTG GCA |
| IL-1β | GAA ATG CCA CCT TTT GAC AGT GAT G | GCT TCT CCA CAG CCA CAA TGA |
| ADORA2A | CAT CCC ATT CGC CAT CAC CA | AAG CCA TTG TAC CGG AGT GG |
| SOCS3 | GAG AGC GGA TTC TAC TGG AGC | ATG CGT AGG TTC TTG GTC CC |
| Bax | AAC TTC ACA GGT TGG CAT TAG G | TTC TTC CAG ATG GTG AGC GAG |
| Bcl-2 | CTT TGA GTT CGG TGG GGT CAT | GCC AGG AGA AAT CAA ACA GAG G |

CSF1, Colony Stimulating Factor 1; IL-6, Interleukin 6; STAT1, Signal Transducer and Activator Of Transcription 1; PTPN6, Protein Tyrosine Phosphatase Non-Receptor Type 6; RAC2, Rac Family Small GTPase 2; TNFα, Tumor Necrosis Factor alpha; IL-1α, Interleukin 1 alpha; IL-1β, Interleukin 1 beta; ADORA2A, adenosine A_2A_ receptor; SOCS3, Suppressor Of Cytokine Signaling 3; Bax, BCL2 Associated X, Apoptosis Regulator; Bcl-2, BCL2 Apoptosis Regulator.
