# Supplementary material for: Therapeutic effects of polydeoxyribonucleotide in an in vitro neuronal model of ischemia/reperfusion injury
Source: Sci Rep. 2023 Apr 12;13:6004. doi: 10.1038/s41598-023-32744-9 (PMC10097812; doi:10.1038/s41598-023-32744-9)
Supplement: Supplementary file 3 — Supplementary Information 3. [file 41598_2023_32744_MOESM3_ESM.pdf]

CSF1, IL-6

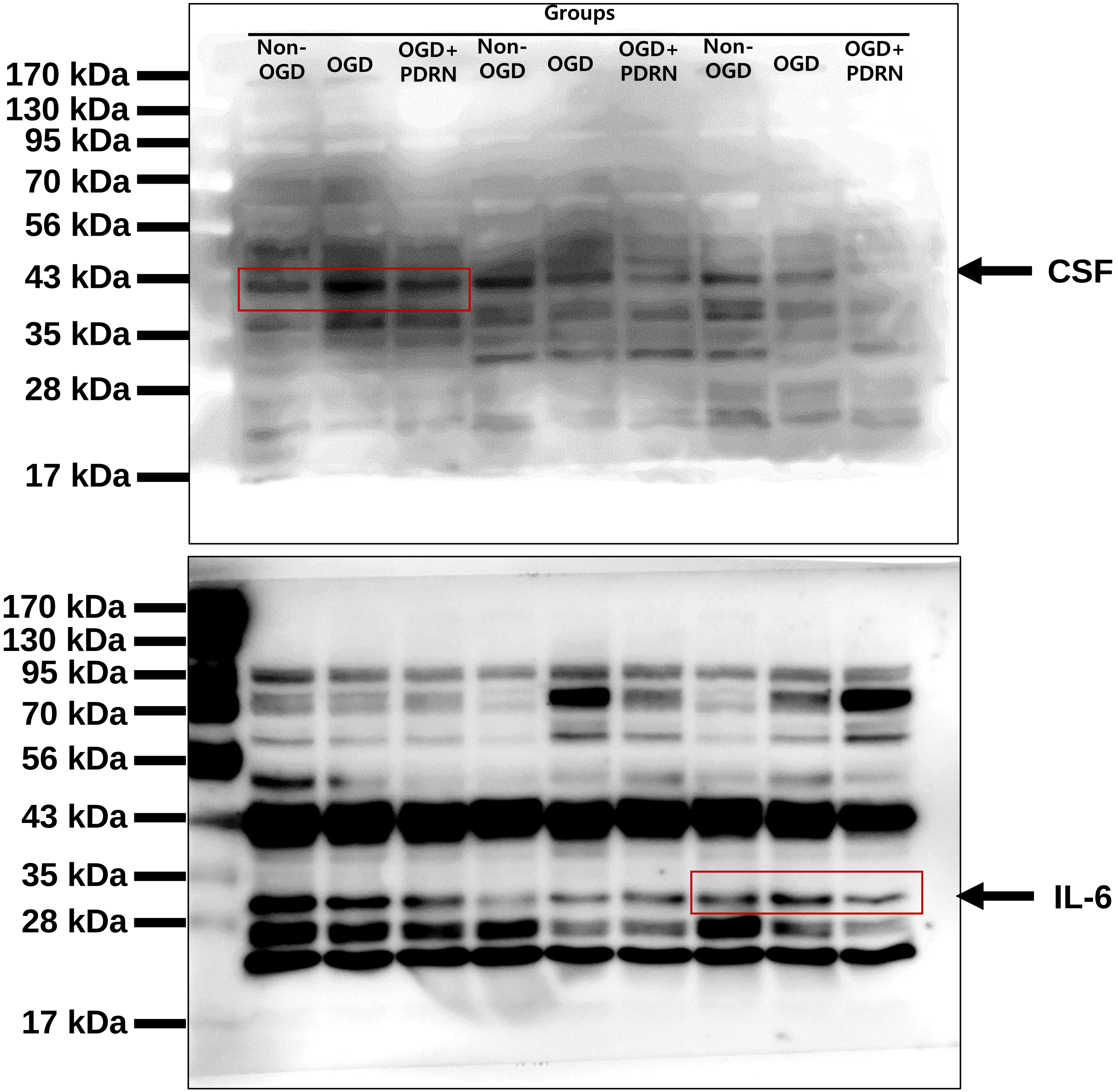

Groups
Non-OGD
OGD
OGD+PDRN
Non-OGD
OGD
OGD+PDRN
Non-OGD
OGD
OGD+PDRN
170 kDa
130 kDa
95 kDa
70 kDa
56 kDa
43 kDa
35 kDa
28 kDa
17 kDa
CSF
170 kDa
130 kDa
95 kDa
70 kDa
56 kDa
43 kDa
35 kDa
28 kDa
17 kDa
IL-6

PTPN6, RAC2

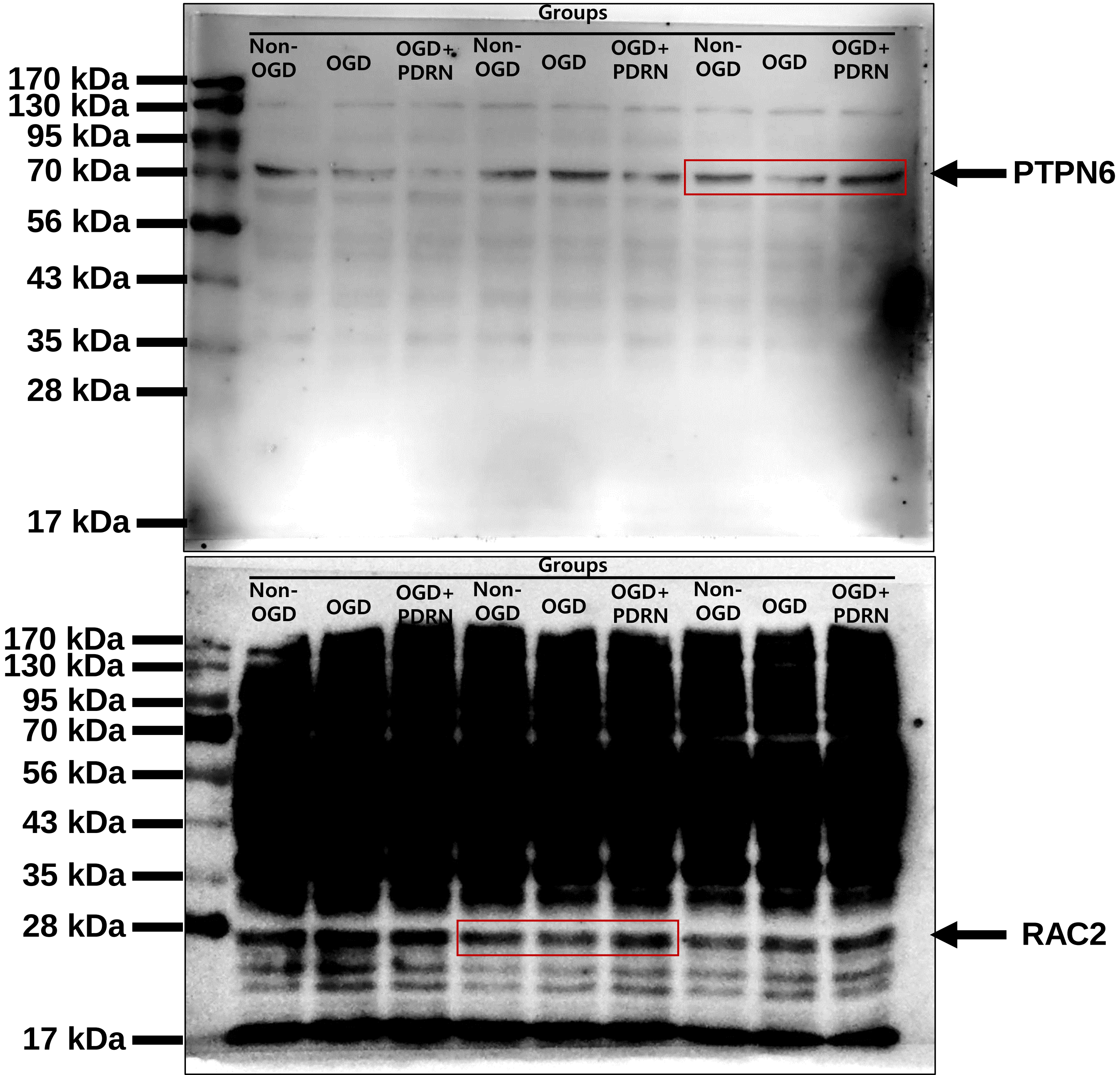
Groups
Non-OGD
OGD
OGD+PDRN
Non-OGD
OGD
OGD+PDRN
Non-OGD
OGD
OGD+PDRN
170 kDa
130 kDa
95 kDa
70 kDa
56 kDa
43 kDa
35 kDa
28 kDa
17 kDa
PTPN6
Groups
Non-OGD
OGD
OGD+PDRN
Non-OGD
OGD
OGD+PDRN
Non-OGD
OGD
OGD+PDRN
170 kDa
130 kDa
95 kDa
70 kDa
56 kDa
43 kDa
35 kDa
28 kDa
17 kDa
RAC2

TNFα, IL-1α

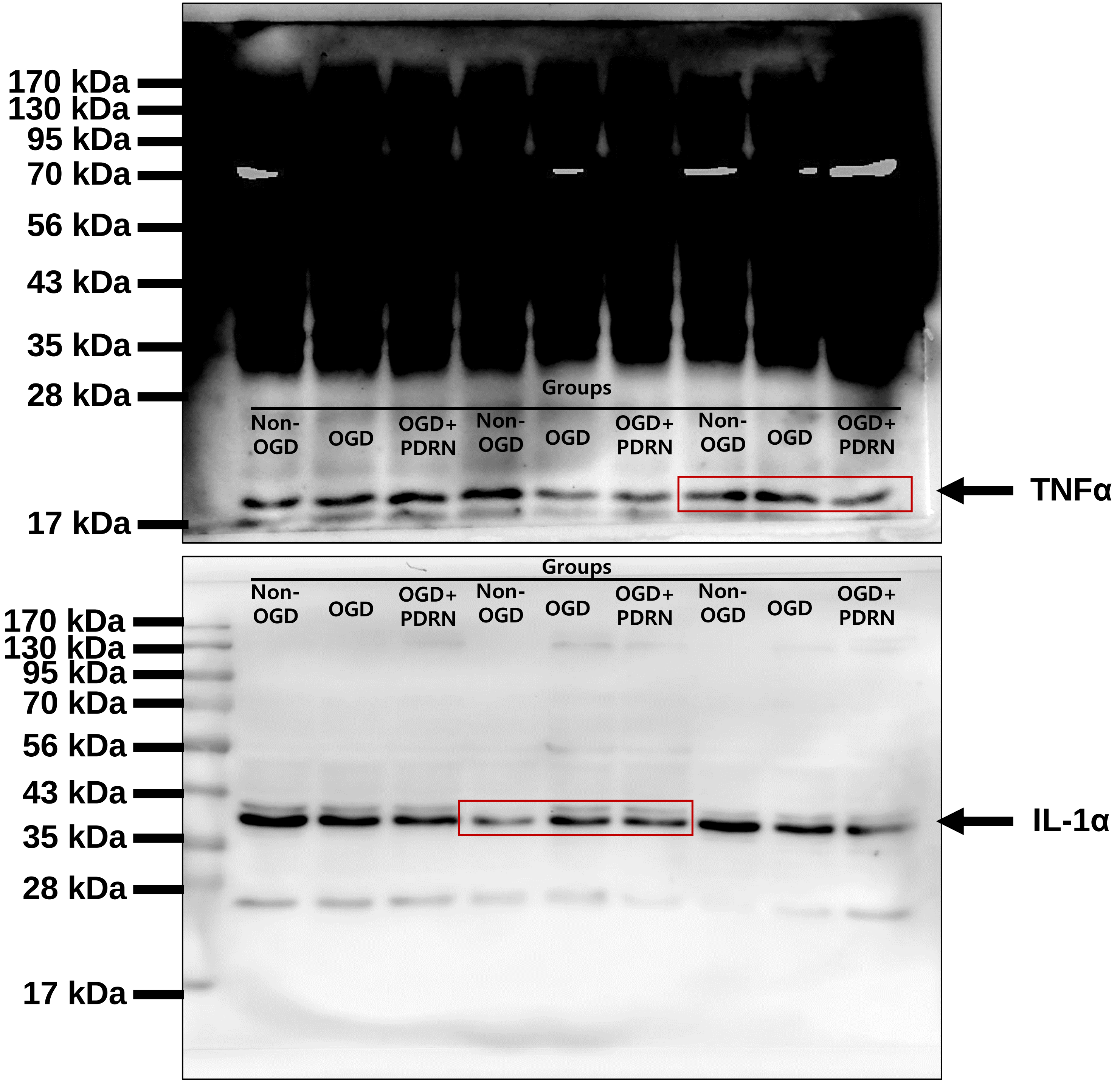

170 kDa
130 kDa
95 kDa
70 kDa
56 kDa
43 kDa
35 kDa
28 kDa
17 kDa
Groups
Non-OGD
OGD
OGD+PDRN
Non-OGD
OGD
OGD+PDRN
Non-OGD
OGD
OGD+PDRN
TNFα
Groups
Non-OGD
OGD
OGD+PDRN
Non-OGD
OGD
OGD+PDRN
Non-OGD
OGD
OGD+PDRN
170 kDa
130 kDa
95 kDa
70 kDa
56 kDa
43 kDa
35 kDa
28 kDa
17 kDa
IL-1α

IL-1β

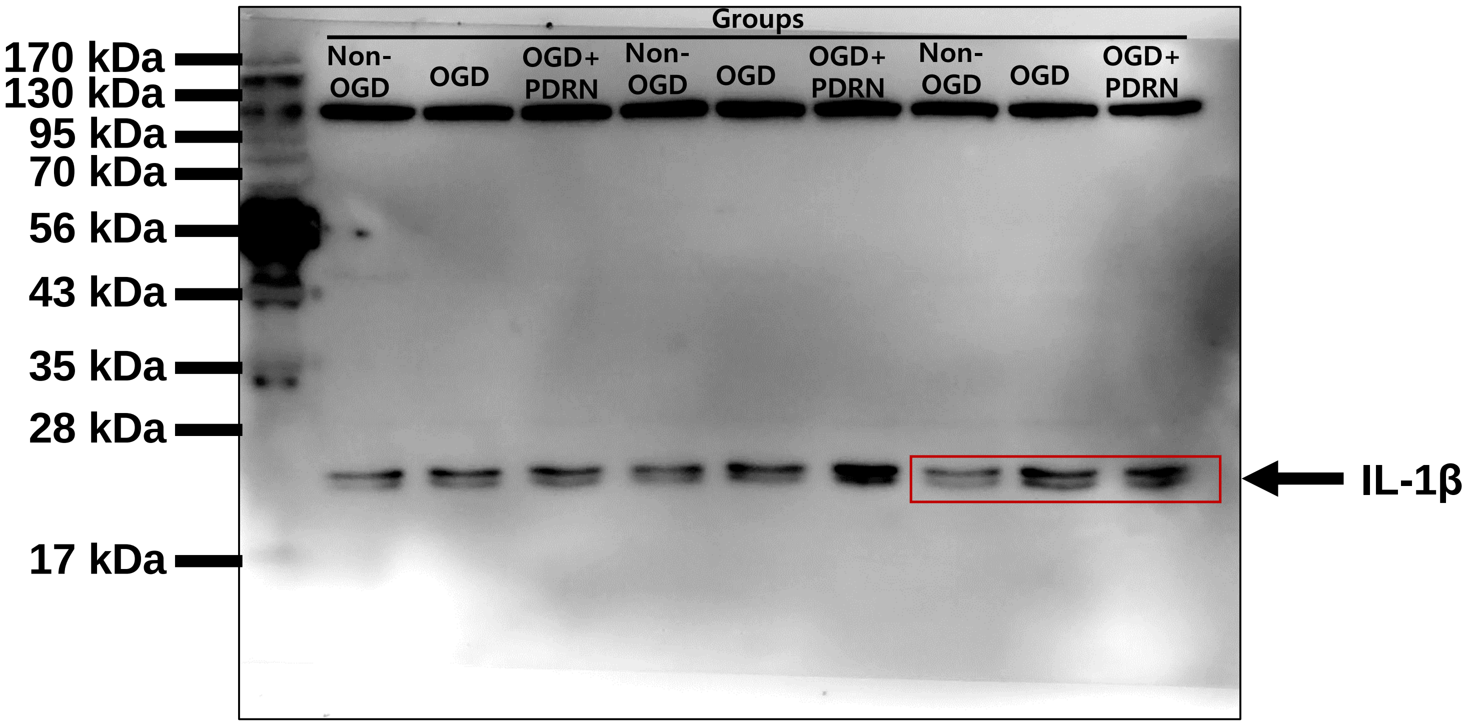
170 kDa
130 kDa
95 kDa
70 kDa
56 kDa
43 kDa
35 kDa
28 kDa
17 kDa
Groups
Non-OGD
OGD
OGD+PDRN
Non-OGD
OGD
OGD+PDRN
Non-OGD
OGD
OGD+PDRN
IL-1β

β-actin

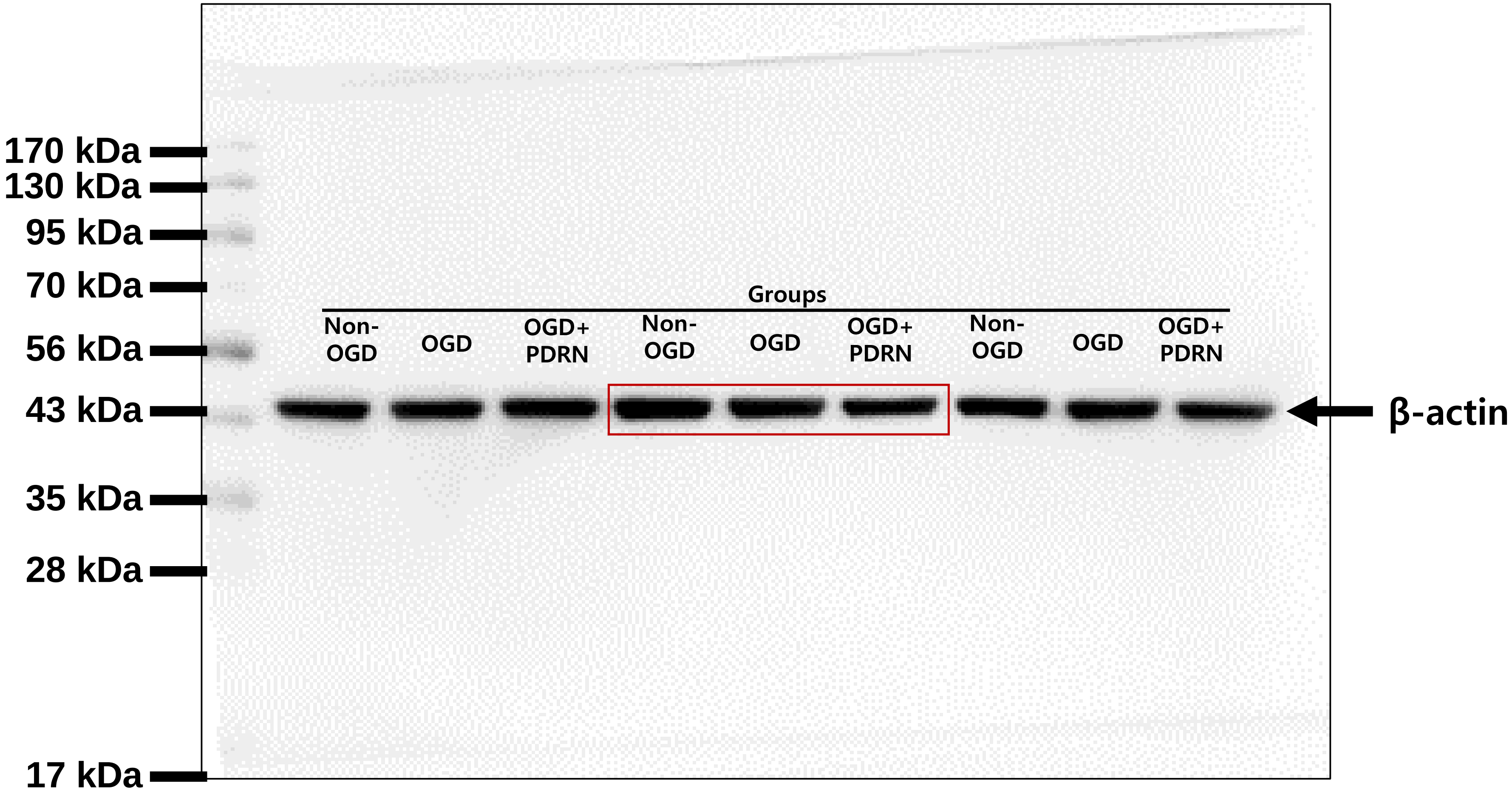

170 kDa
130 kDa
95 kDa
70 kDa
56 kDa
43 kDa
35 kDa
28 kDa
17 kDa
Groups
Non-OGD
OGD
OGD+PDRN
Non-OGD
OGD
OGD+PDRN
Non-OGD
OGD
OGD+PDRN
β-actin

ADORA2A, SOCS3

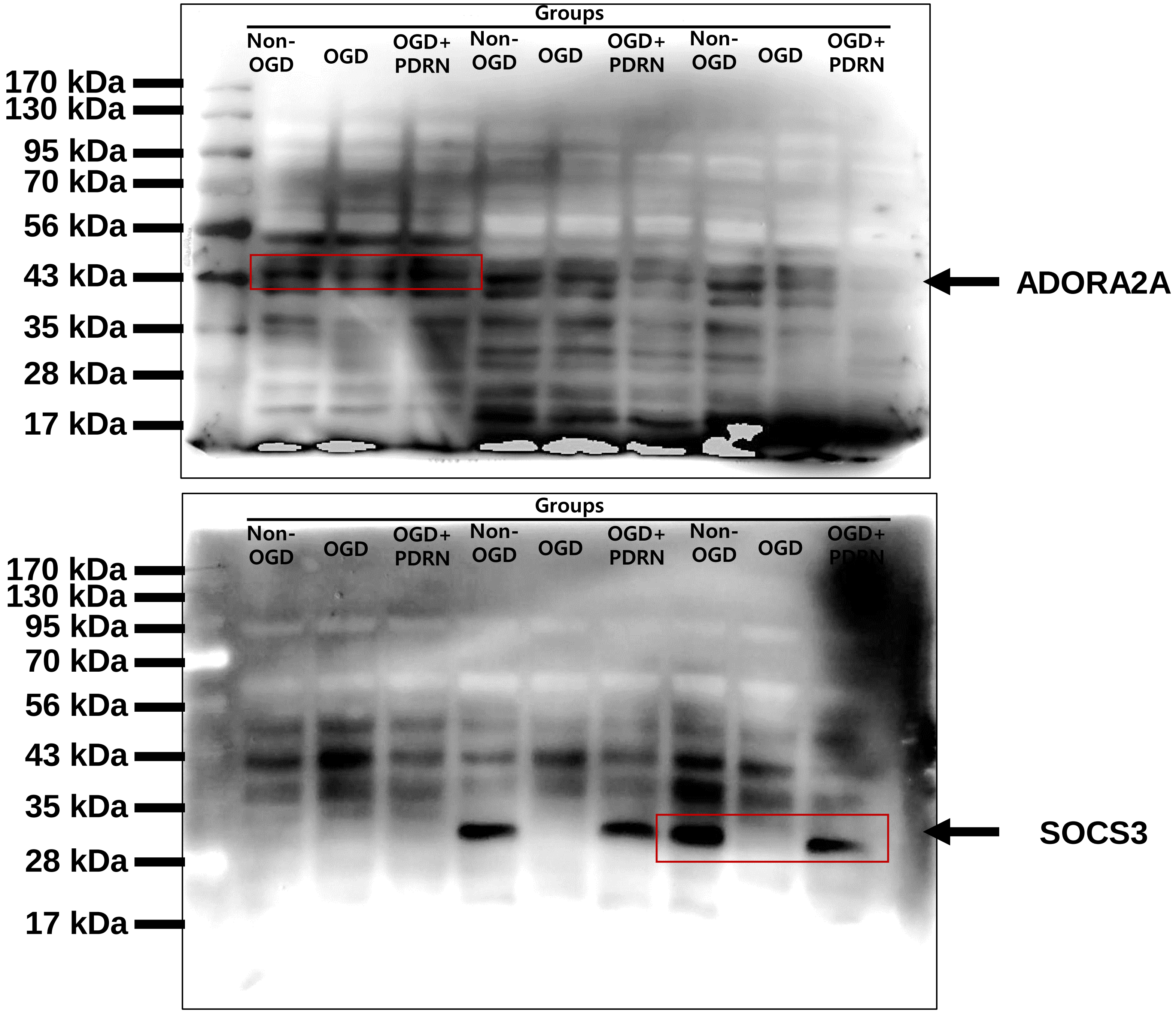

p-JAK1, JAK1

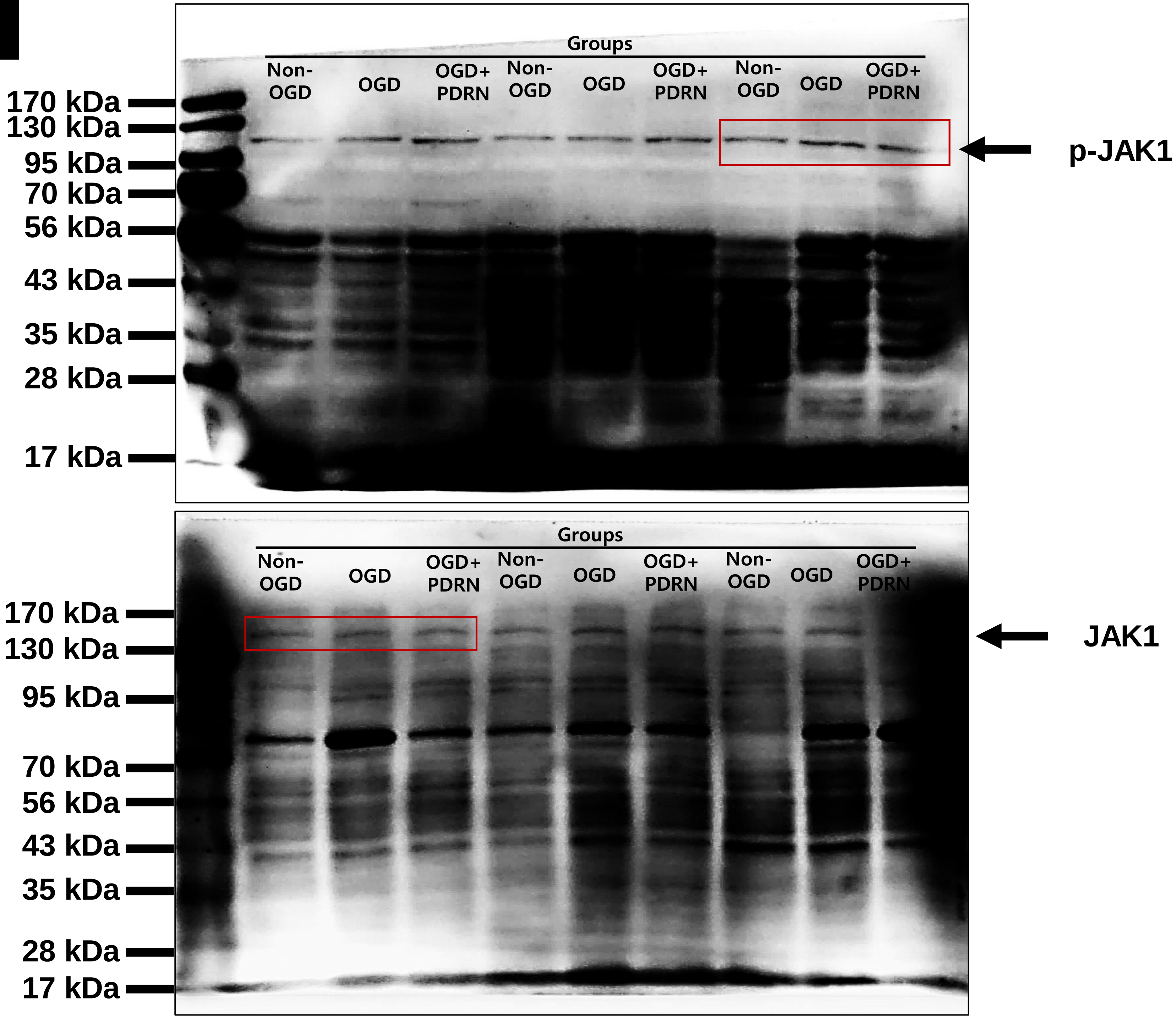

Groups
Non-OGD
OGD
OGD+PDRN
Non-OGD
OGD
OGD+PDRN
Non-OGD
OGD
OGD+PDRN
170 kDa
130 kDa
95 kDa
70 kDa
56 kDa
43 kDa
35 kDa
28 kDa
17 kDa
p-JAK1
Groups
Non-OGD
OGD
OGD+PDRN
Non-OGD
OGD
OGD+PDRN
Non-OGD
OGD
OGD+PDRN
170 kDa
130 kDa
95 kDa
70 kDa
56 kDa
43 kDa
35 kDa
28 kDa
17 kDa
JAK1

p-JAK2, JAK2

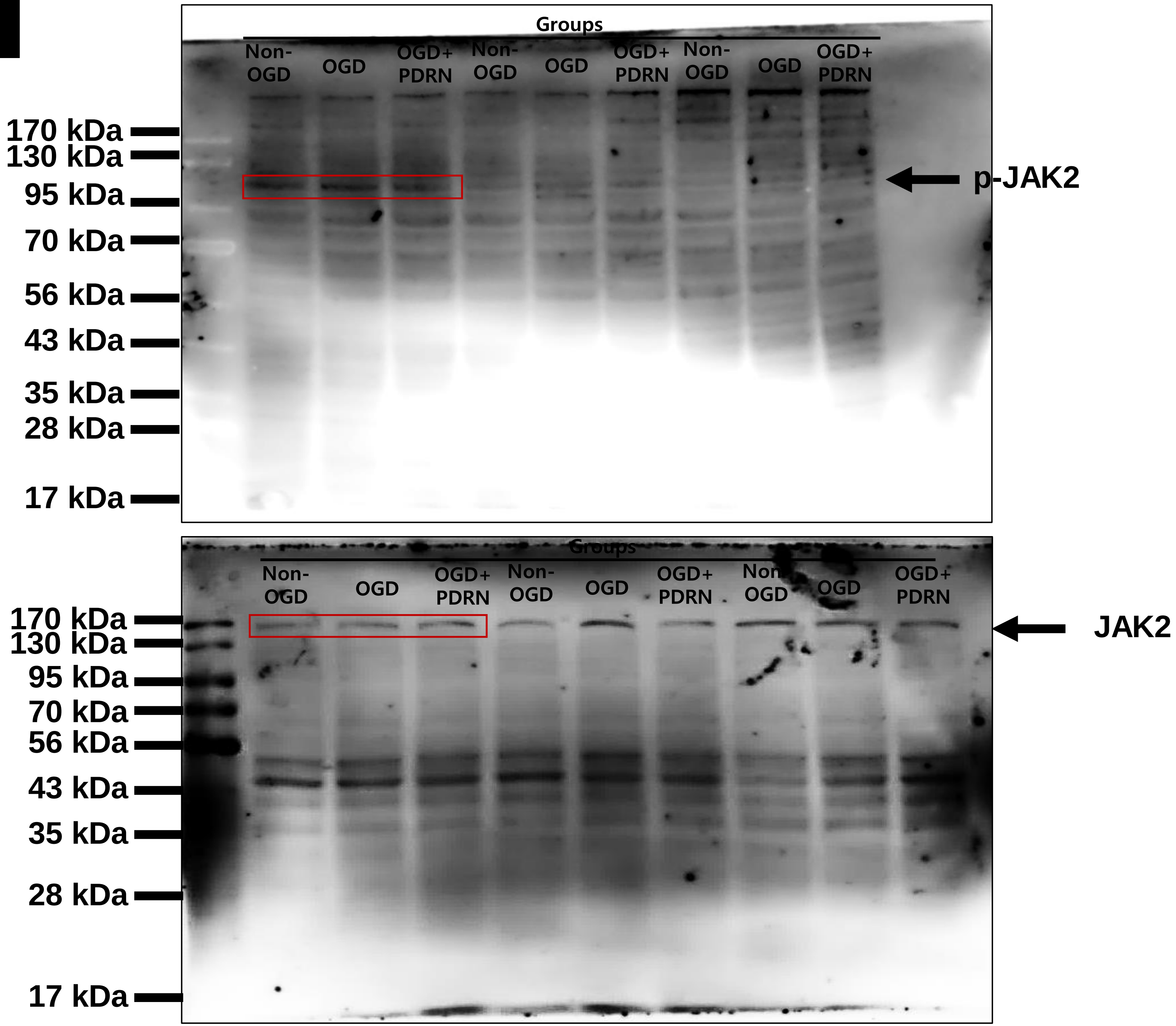
Groups
Non-OGD
OGD
OGD+PDRN
Non-OGD
OGD
OGD+PDRN
Non-OGD
OGD
OGD+PDRN
170 kDa
130 kDa
95 kDa
70 kDa
56 kDa
43 kDa
35 kDa
28 kDa
17 kDa
p-JAK2
Groups
Non-OGD
OGD
OGD+PDRN
Non-OGD
OGD
OGD+PDRN
Non-OGD
OGD
OGD+PDRN
170 kDa
130 kDa
95 kDa
70 kDa
56 kDa
43 kDa
35 kDa
28 kDa
17 kDa
JAK2

p-STAT1, STAT1

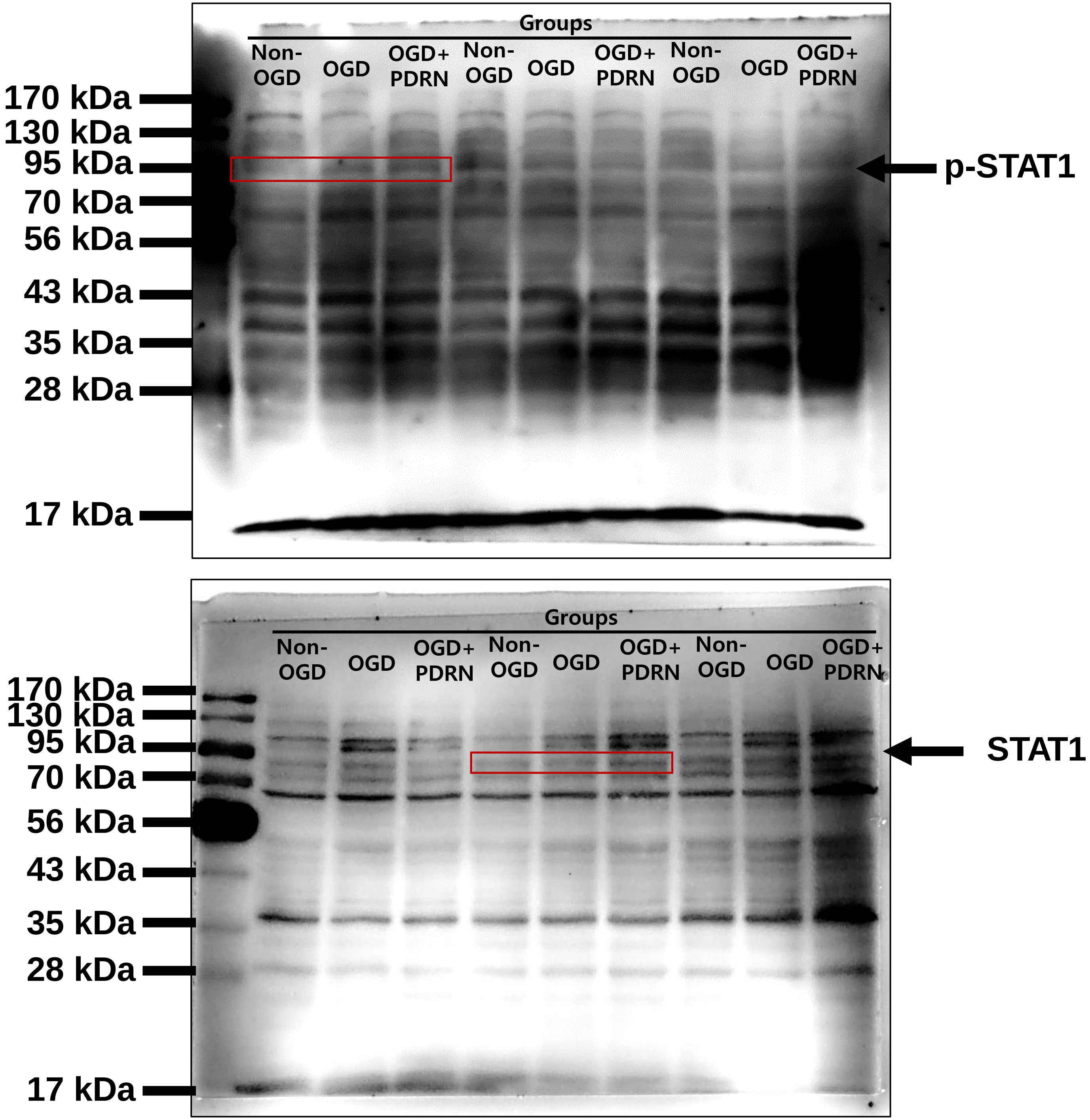

p-STAT3, STAT3

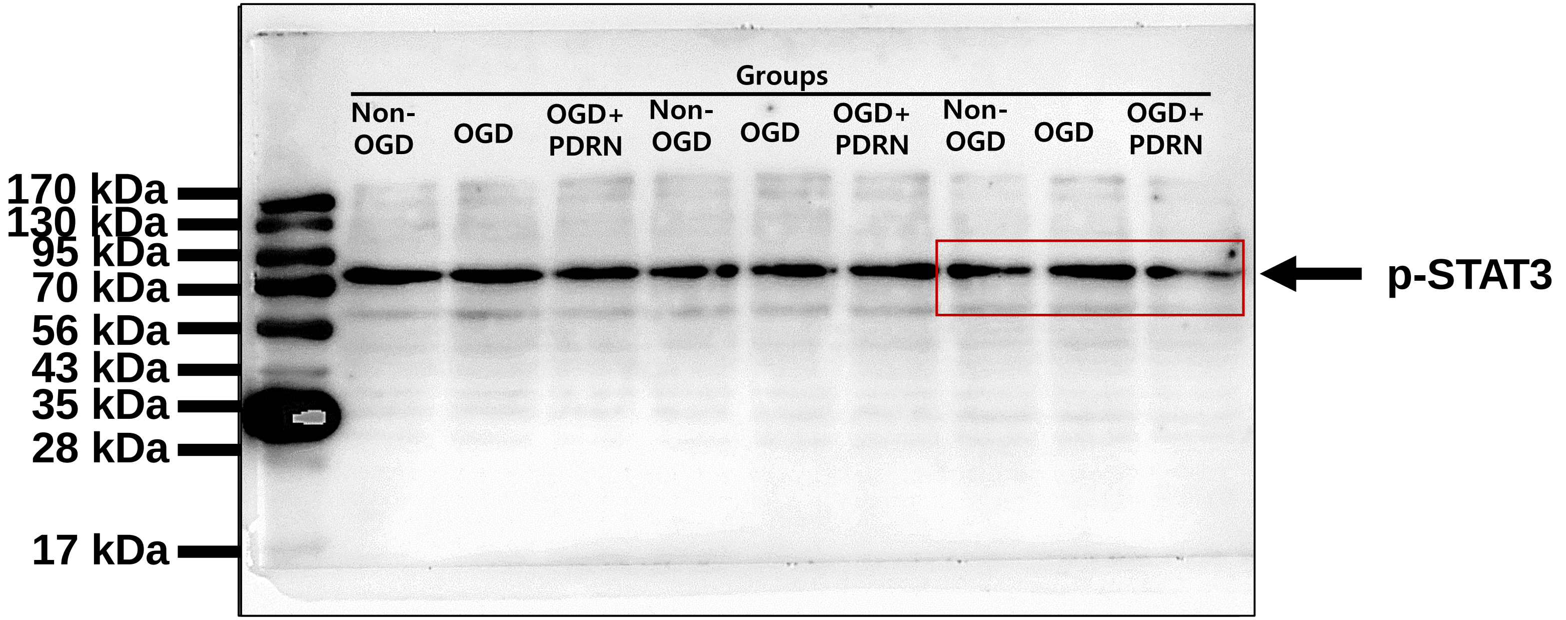

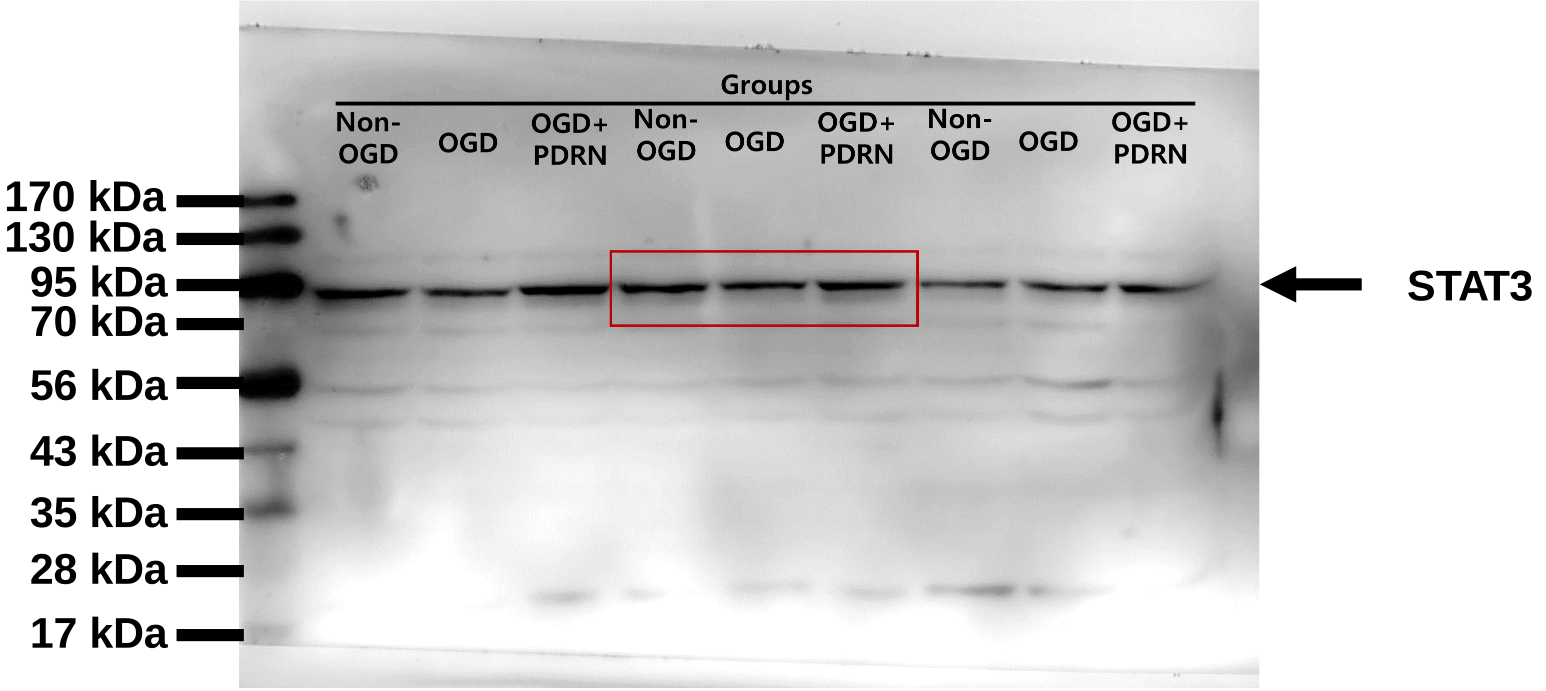

β-actin

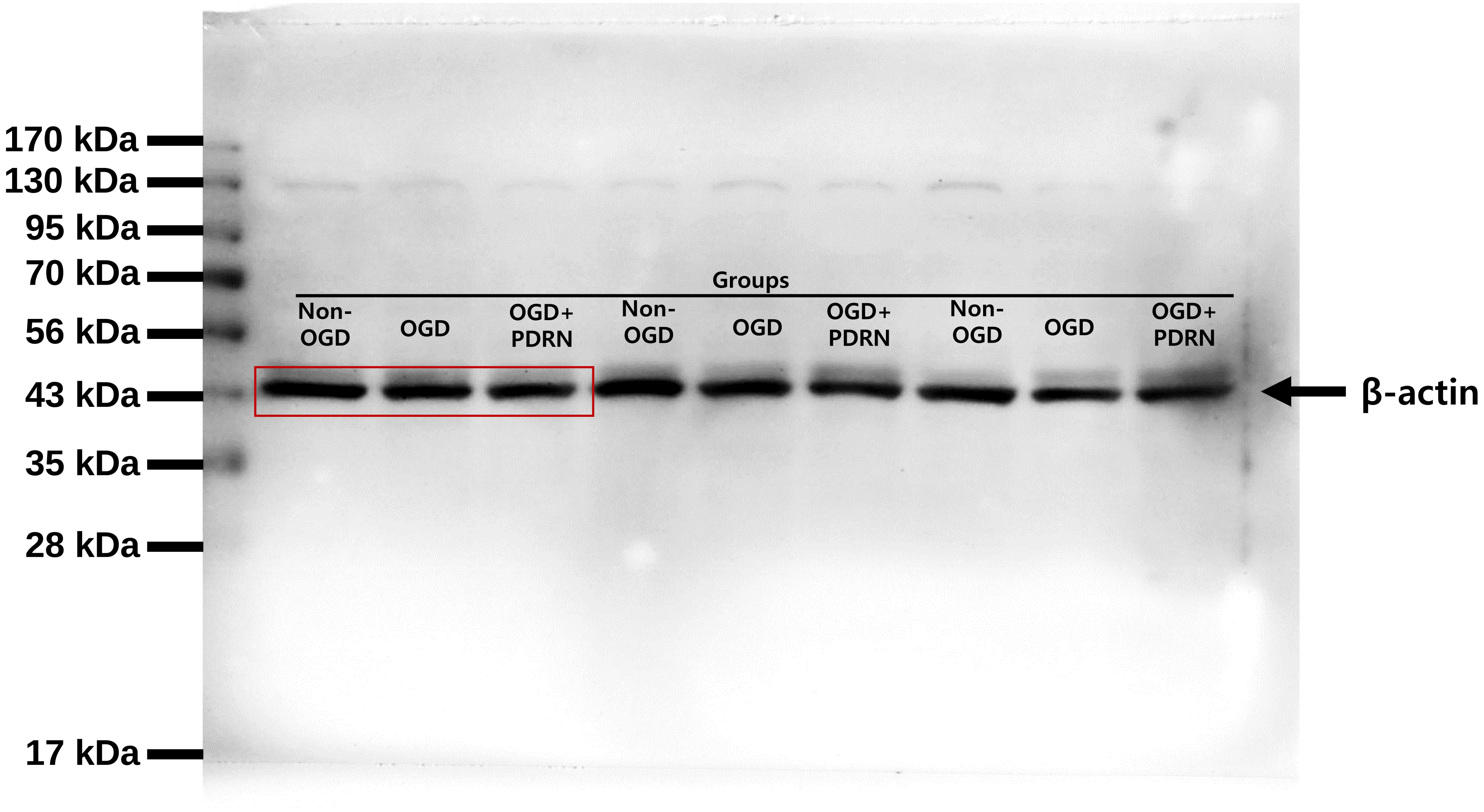

170 kDa
130 kDa
95 kDa
70 kDa
56 kDa
43 kDa
35 kDa
28 kDa
17 kDa
Groups
Non-OGD
OGD
OGD+PDRN
Non-OGD
OGD
OGD+PDRN
Non-OGD
OGD
OGD+PDRN
β-actin

Bax, Bcl-2

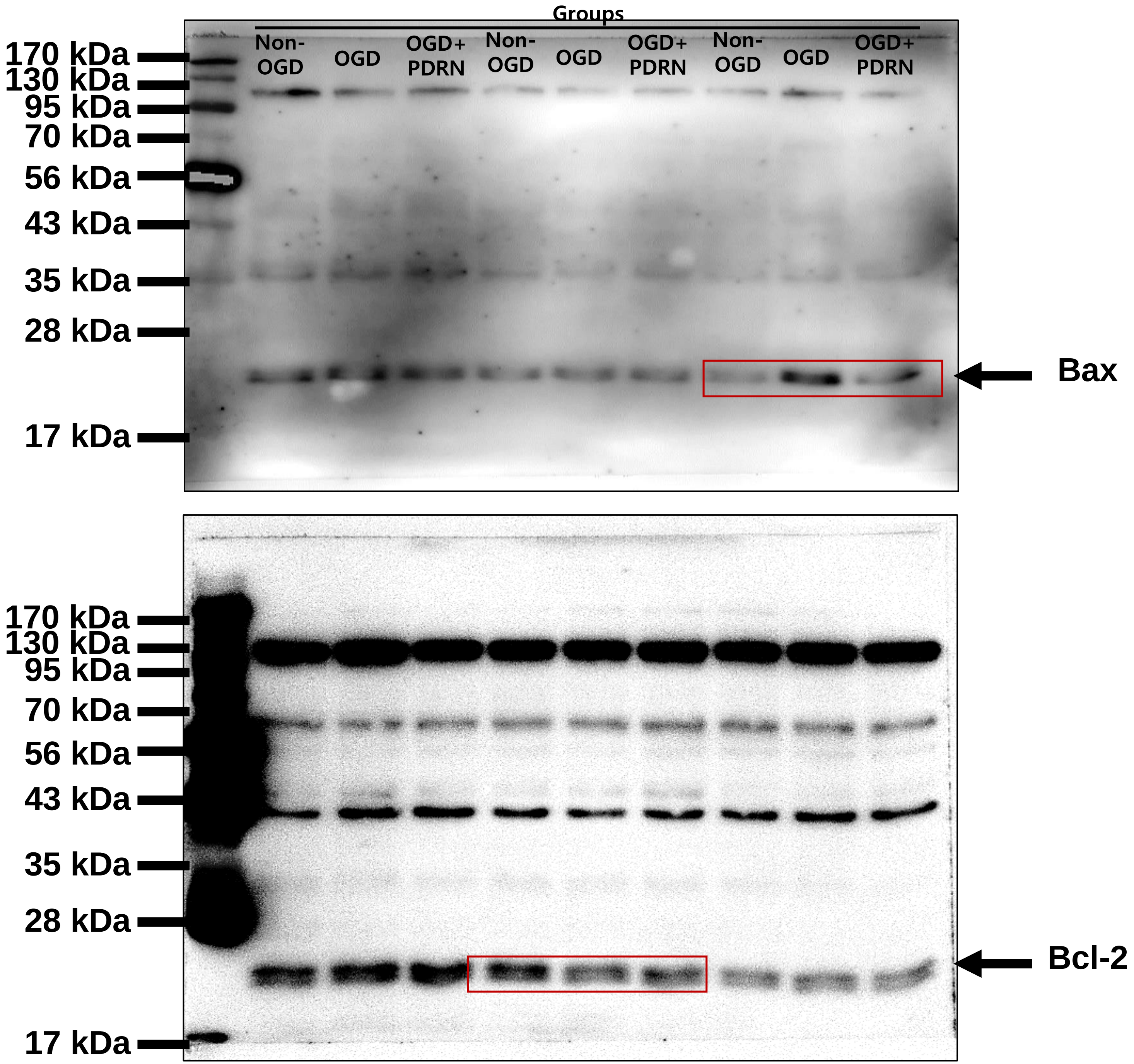
Groups
Non-OGD
OGD
OGD+PDRN
Non-OGD
OGD
OGD+PDRN
Non-OGD
OGD
OGD+PDRN
170 kDa
130 kDa
95 kDa
70 kDa
56 kDa
43 kDa
35 kDa
28 kDa
17 kDa
Bax
170 kDa
130 kDa
95 kDa
70 kDa
56 kDa
43 kDa
35 kDa
28 kDa
17 kDa
Bcl-2

β-actin

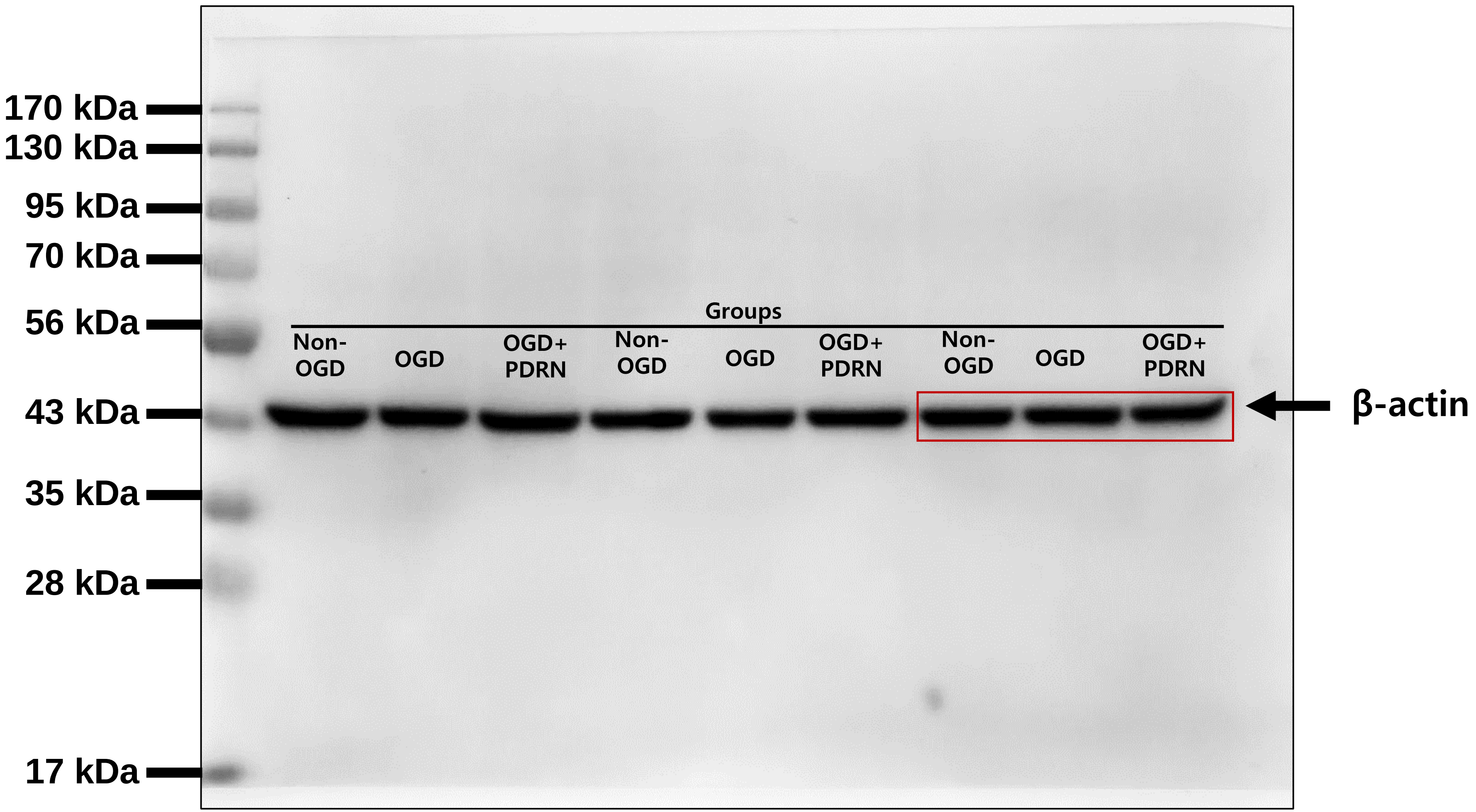
170 kDa
130 kDa
95 kDa
70 kDa
56 kDa
43 kDa
35 kDa
28 kDa
17 kDa
Groups
Non-OGD
OGD
OGD+PDRN
Non-OGD
OGD
OGD+PDRN
Non-OGD
OGD
OGD+PDRN
β-actin
